# Identification of a putative novel rotavirus A VP4 genotype in bats from an urban Atlantic Forest, in the metropolitan region of Rio de Janeiro State, Southeastern Brazil

**DOI:** 10.1007/s00705-026-06727-w

**Published:** 2026-10-08

**Authors:** Beatriz Vilete Dias, Joana Medina Maçaira, Yan Cardoso Pimenta, Meylin Bautista Gutierrez, Maria Ogrzewalska, Ricardo Moratelli, Roberto Leonan Morim Novaes, Marina Carvalho Furtado, Sócrates Fraga Costa-Neto, Juliana da Silva Ribeiro de Andrade, Tulio Machado Fumian, José Paulo Gagliardi Leite, Johan Nordgren, Marcia Terezinha Baroni de Moraes, Marina Galvão Bueno

**Affiliations:** 1 Laboratory of Comparative and Environmental Virology (LVCA), Oswaldo Cruz Institute (IOC), Oswaldo Cruz Foundation (FIOCRUZ), Rio de Janeiro, RJ Brazil; 2 Laboratory of Respiratory Viruses, Exanthematics, Enteroviuses and Viral Emergencies (LVRE), Oswaldo Cruz Institute (IOC), Oswaldo Cruz Foundation (FIOCRUZ), Rio de Janeiro, RJ Brazil; 3 Fiocruz Mata Atlântica, Oswaldo Cruz Foundation (FIOCRUZ), Rio de Janeiro, RJ Brazil; 4 Department of Science and Technology, Ministry of Health of Brazil, Brasília, DF Brazil; 5 Linköping University, Linköping, 581 85 Sweden

**Keywords:** Chiroptera, *Rotavirus alphagastroenteritidis*, Novel genotype, Interspecies transmission, Surveillance

## Abstract

Cross-species transmission occurs in rotaviruses, and human infection by animal strains is possible. We investigated the occurrence and genetic diversity of Rotavirus A (RVA) in bats from urban Atlantic Forest remnants under intense anthropogenic pressure in Rio de Janeiro, Brazil. Bats were captured between September 2020 and March 2022, totaling 240 samples (rectal swabs and feces) from 16 species. The RVA infection rate was 1.25% (3/240; *Artibeus lituratus and Carollia perspicillata*) using RT-qPCR targeting the NSP3 gene. A complete genomic constellation was attempted for positive samples. Phylogenetic analyses were performed, and homology-based three-dimensional modeling was applied to proteins encoded by genes showing low similarity to GenBank sequences. Partial VP4 sequences were successfully obtained from all three RVA-positive samples. Molecular characterization identified distinct genotypes: Gx-P[26]-I5, G20-P[unclassified*]-I13-T15, and G20-P[unclassified*]-I13-R13-C13-M12-A23-N13-T15-Ex-H15. The latter two harbored a putative novel VP4 genotype (P[unclassified*]) with <75% nucleotide identity to known genotypes and formed a distinct, well-supported phylogenetic clade. The Gx-P[26]-I5 strain showed highest identity to porcine RVA strains, representing the first detection of VP4 genotype P[26] in bats. Except for VP4, both G20P[unclassified*] strains were most closely related to a human G20P[28] strain from Suriname, suggesting a shared genomic constellation and possible reassortment between bat and human viruses. 3D structural analyses revealed conserved VP4 conformation among animal and human strains, suggesting structural compatibility across host species. These findings highlight bats as reservoirs of diverse RVA lineages and their role in viral evolution, reassortment, and zoonotic emergence, reinforcing the need for continuous One Health surveillance.

**Supplementary Information:**

The online version contains supplementary material available at https://doi.org/10.1007/s00705-026-06727-w.

## Introduction

Over the past two decades, there has been an increased focus on One Health approach, that highlights the interconnection between human, animal, plants and environmental health [1]. In this context, monitoring the health, biodiversity, and epidemiological status of wildlife, particularly in areas subject to human disturbance and near expanding urban zones, is essential for guiding both conservation initiatives and public health programs [2].

Bats are particularly important animals for pathogen surveillance, as they are recognized hosts of a broad diversity of zoonotic viruses [3]. Their high adaptability enables them to occupy both urban environments and forests adjacent to densely populated human areas, making them key players in the maintenance and transmission of viruses with zoonotic potential, such as coronaviruses, rabies virus, paramyxoviruses and adenoviruses [4–7]. With over 1,460 species described worldwide, including 186 species described in Brazil, bats represent the second largest mammalian group in terms of viral richness with zoonotic potential [8–10]. Although bats can carry pathogens, they play crucial ecological roles in insect control, plant pollination, and seed dispersal, and act as important bioindicators of environmental change, especially in habitats affected by human activity [11–13].

Viruses associated with gastroenteric diseases in animals and humans are of particular importance, and rotaviruses cause acute gastroenteritis (AGE) in a wide range of mammals, including humans and livestock species [14]. Rotaviruses belong to the family *Sedoreoviridae* and are characterized by an icosahedral symmetry and a non-enveloped structure. *Rotavirus alphagastroenteritidis* (formerly Rotavirus A, RVA) possesses a segmented double-stranded RNA (dsRNA) genome encoding six structural proteins (VP1–VP6) and six non-structural proteins (NSP1–NSP6) [15]. There are 11 recognized rotavirus species and, RVA is the most epidemiologically significant, being a major cause of AGE morbidity and mortality in children < 5 years old worldwide and animals, and the biggest public health treat [16–22]. RVA is highly stable under environmental and gastrointestinal conditions and is mainly transmitted via the fecal–oral route. Its high infectivity, extensive shedding, and resistance to physical and chemical inactivation promote environmental persistence and dissemination, particularly in anthropized environments where close contact among humans, domestic animals, and wildlife may facilitate interspecies transmission [18–22].

RVA has a binary classification based on two outer capsid proteins that induce neutralizing antibodies: VP7 (“G” genotype - glycoprotein) and VP4 (“P” genotype - protease-sensitive). While binary classification is standard for epidemiology, a complete genome classification system (gene constellation) is fundamental to identify RVA reassortments and viruses’ evolution. Consequently, a new RVA classification based on nucleotide identity across the 11 genome segments was proposed, forming the genomic constellation: Gx–P[x]–Ix–Rx–Cx–Mx–Ax–Nx–Tx–Ex–Hx (VP7–VP4–VP6–VP1–VP2–VP3–NSP1–NSP2–NSP3–NSP4–NSP5) [23–25] which supports comparative analyses of genetic diversity, reassortment, and viral evolution. To date, numerous genotypes have been reported in humans and animals, including at least 42 G and 58 P genotypes, according to Rotavirus Classification Working Group (RCWG) [14, 18, 24, 26, 27].

In bat populations, 23 *Rotavirus alphagastroenteritidis* RVA genomic constellations (18 complete and five partial) have been described in wild populations [14]. These have been reported across all continents, including the Americas, where genotypes such as G20-P[x], G3-P[3]-T3-E3-H6, Gx-P[4], Gx-P[6] and G20-P[x]-T15-H15, have been identified in Brazil [26, 27] and G20-P [47]-I13-R13-C13-M12-A32-N13-T23-E23-Hx in Costa Rica [18].

Given the capacity of RVA for interspecies transmission and to undergo genetic reassortment between human and animals’ strains, generating novel genotypes with potential to spread between humans [18, 26, 34], continuous surveillance of circulating RVA genotypes in wildlife, including bats, is important. Additionally, there are very limited data on RVA investigation in bats samples from Brazil. Therefore, this study aimed to detect and genetically characterize RVA circulation in bat populations inhabiting one of the largest remaining urban Atlantic Forest areas in the metropolitan region of Rio de Janeiro, Brazil. For this purpose, faecal samples collected from bats were screened using RT-qPCR, and positive samples were sequenced to determine their genotype constellation. Our findings suggest the possible identification of novel genotypes and provide insights into potential interspecies transmission events within a One Health framework.

## Materials and methods

### Study area

**Fig. 1:**
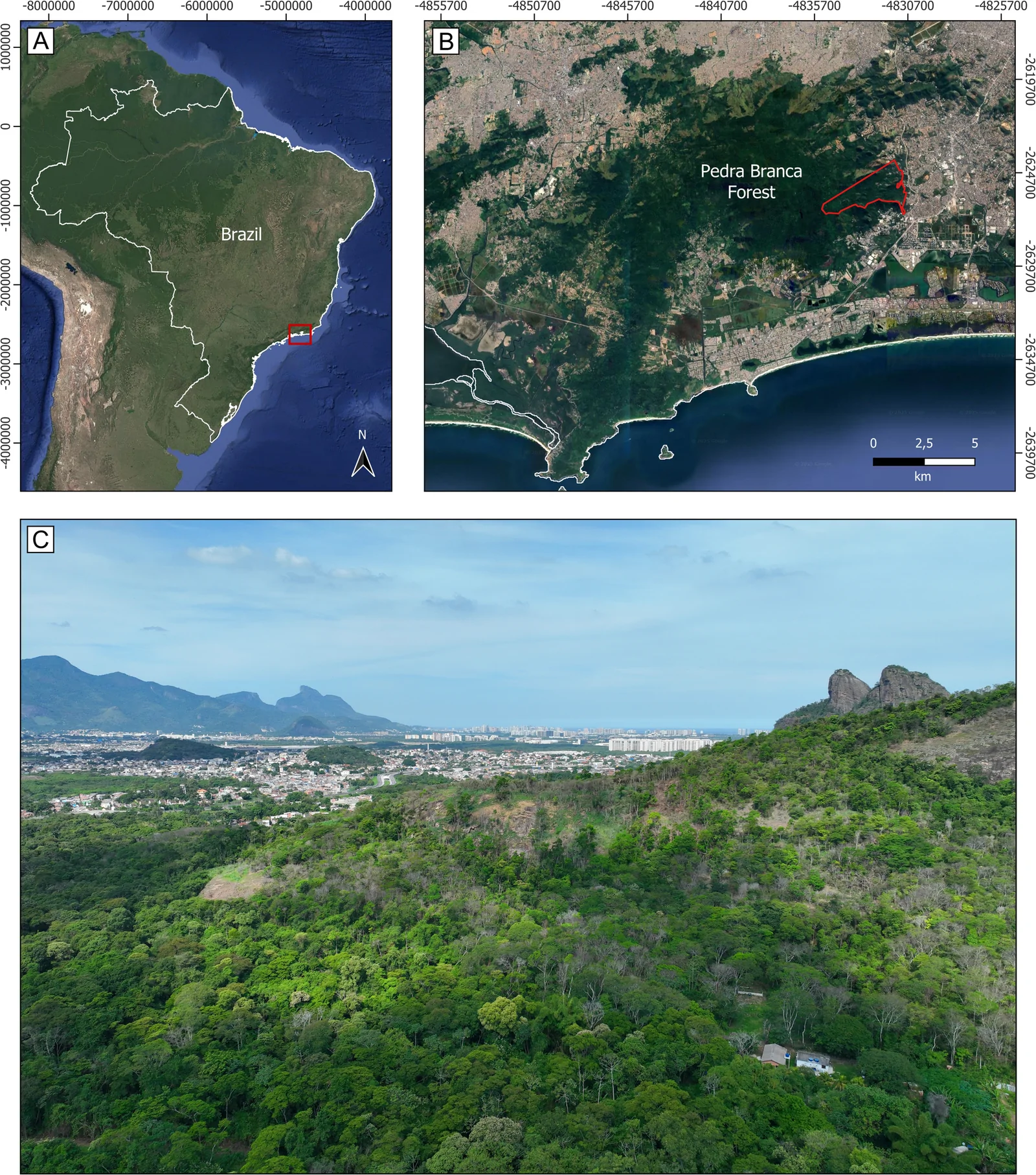
Sampling sites of bats positive for Rotavirus A, in an urban Atlantic Forest remnant, Rio de Janeiro, Brazil, September 2020–March 2022. **A** Map of Brazil highlighting the state of Rio de Janeiro in red. Map of the Rio de Janeiro metropolitan region showing the location of the Pedra Branca Forest (PBF) and the bat sampling site (circled in red). **B** Close-up image of the Atlantic Forest area where bats were captured, illustrating the extensive surrounding metropolitan area; the PBF is one of the largest urban forests in the Americas. Image: Google Earth website. http://earth.google.com/, 2025

The study was carried out in Pedra Branca Forest (PBF), metropolitan region of Rio de Janeiro State, Southeastern Brazil, that is considered one of the largest remaining urban forests in the world with 462 hectares. Animal samples were collected within the PBF at the Fiocruz Atlantic Forest Biological Station (22°56′25″S, 43°24′18″W), an area located on the eastern slope of the PBF (Fig. 1). Over 12.000 ha of tropical rainforest vegetation represent the PBF, varying between pristine forest areas and areas integrated to extremely urbanized landscapes. These urbanized zones are formed by densely human populated neighbourhoods with extensive social vulnerability and lack of proper sanitation, which highlights the probable impact of the encounters that happen between wild, domestic animals and humans in this region [30].

### Animal handling and sample collection

Bats were captured between September 2020 and March 2022 using 10 mist nets (3 × 9 m, 15 mm mesh), placed before dusk within the potential flight paths of the studied species [31]. The nets were checked for fallen animals within every 20 min, then taken off after a total of five hours, comprehending the period of peak activity of most species. For the study, 35 nights of collecting and sampling were conducted, divided into 10 capture campaigns. After being captured, the animals were physically restrained and biometrics measures were evaluated, followed by sex determination, species identification, age category estimation and reproductive status assessment. Rectal swabs (FLOQSwabs^®^ Flocked Swabs, 518CS01) and faecal samples were collected from each individual and immediately placed in 1 mL of in-house Viral Transport Medium (VTM), following a previously described protocol [6], with faecal samples collected opportunistically during handling. Some rectal swab samples were stored together with faecal material in the same cryotube, whereas in other cases faecal samples were stored separately from the swabs. Samples were kept on ice in the field and subsequently stored at − 80 °C until analysis. Sterile materials and personal protective equipment were used throughout to prevent cross-contamination. A portion of the bats was returned to their natural habitat after sample collection, at the same capture location (224/240), and another portion was euthanized (16/240) by exsanguination under deep anaesthesia, for use in other studies from a broader biodiversity project, in which this work is included.

### Ethics approvals

The procedures for animal capture and handling were conducted in strict compliance with the bioethics and biosafety guidelines established by the Fiocruz Biosafety Committee. This study was duly approved by the Fiocruz Ethics Commission on Animal Use (CEUA-Fiocruz LM-6/18 and LW-18/23). Authorization for the collection and transportation of all samples was obtained from the Brazilian Institute of the Environment and Natural Renewable Resources (SISBIO No. 19037–1) and the National System for the Management of Genetic Heritage and Associated Traditional Knowledge (SISGEN A46BOE1 and A918B14).

### Molecular assays and analyses

#### RVA detection

Rectal swab and faecal samples collected from bats underwent nucleic acid extraction using the QIAamp Viral RNA Mini Kit (Qiagen), following the manufacturer’s protocol. RVA detection was carried out by RT-qPCR as previously described [29, 35], using primers and probe targeting the conserved NSP3 gene region of RVA genome, using the SuperScript™ III Platinum™ One-Step RT-qPCR Kit (Thermo Fisher Scientific, Waltham, MA, USA).

#### Genotyping and nucleotide sequencing

RVA-positive samples were subjected to genome constellation analysis based on partial sequencing of genomic segments using different primer sets designed to amplify each viral gene under specific protocols. The supplementary material (Supplementary Table S1) summarizes the primer sets and RT-PCR assays used for the amplification of the target genes. All primers employed for RVA genotyping were selected based on previously published studies [33–37]. Initially, RT-PCR was performed using the SuperScript™ III One-Step RT-PCR System with Platinum™ Taq DNA Polymerase (Thermo Fisher Scientific, Waltham, MA, USA) (Supplementary Table S1). If amplification was unsuccessful using the one-step protocol, a two-step approach was employed. Briefly, reverse transcription was first carried out using SuperScript™ III Reverse Transcriptase (Thermo Fisher Scientific, Waltham, MA, USA), followed by PCR amplification with iTaq™ DNA Polymerase (Bio-Rad Laboratories, Hercules, California, USA), under the following the number of cycling and melting temperature varying according to each primers set (Supplementary Table S1).

PCR amplicons were visualized on 1.5% agarose gel stained with SYBR Safe DNA Gel Stain (Thermo Fisher Scientific, Waltham, MA, USA). Samples that failed to produce detectable products in the initial PCR reaction, were subjected to a second round of amplification (booster PCR) [38]. This reaction was performed using the same primer sets and cycling conditions as the first round, with 2 µL of the initial amplicon as template and iTaq™ DNA Polymerase reagents according to the manufacturer’s instructions (Bio-Rad Laboratories, Hercules, CA, USA). Amplicons were purified either with the QIAquick Gel Extraction Kit (Qiagen, Hilden, Germany) or the ExoSAP clean-up system (Thermo Fisher Scientific, Waltham, MA, USA). Sequencing was performed by the Fiocruz sequencing platform using the Nucleotide Sanger method. The resulting nucleotide sequences were processed and analyzed in MEGA X [39] and subsequently compared with available sequences in the NCBI GenBank database using BLASTn searches. Representative sequences generated in this study were deposited in GenBank: PV740368, PV802099 - PV802103, PV827188 - PV827194, PV841819 - PV841821.

#### Phylogenetic analysis

Consensus sequences were generated with Mega-Molecular Evolutionary Genetic Analysis Version X software [39] or Geneious Prime (Biomatters Ltd., Auckland, New Zealand). The RVA genotypes were verified by identifying the closest homologous sequences through the Basic Local Alignment Search Tool (BLAST) and Rotavirus A Genotyping Tool Version 0.1 (https://mpf.rivm.nl/mpf/typingtool/rotavirusa/, accessed on 1 September of 2025). Phylogenetic analyses were conducted using *MEGA X* (v.10.1.7) [39]. Maximum likelihood (ML) analyses were conducted under the best-fit nucleotide substitution model, which was determined separately for each dataset based on model selection criteria and 2.000 bootstrap replicates to assess branch support, using RVA reference sequences retrieved from the National Center for Biotechnology Information (NCBI) database.

#### In silico structural modeling, structural analysis and visualization of the RVA VP4 protein

A partial amino acid sequence of the VP4 protein from RVA identified in this study, together with homologous sequences from representative reference strains retrieved from GenBank (NCBI), was subjected to comparative three-dimensional structural modeling based on homology. The analyzed sequences comprised PQ215632.1 (P[1]; bovine), JF712558.1 (P[18]; equine), OP344997.1 (P[26]; swine), DQ061053.1 (P[26]; swine), MH285840.1 (P[51]; bat), and KX257407.1 (P[28]; human strain from Suriname).

Structural models were generated using the Phyre2 v2.2 server (accessed in February 2026) under standard (“normal”) modeling mode. For each sequence, the top-ranked template identified by the server based on structural homology was selected. Subsequently, the model exhibiting the highest confidence score and maximal sequence coverage was retained for downstream analyses.

Three-dimensional visualization and structural comparisons were performed using PyMOL v3.1.6.1. To ensure consistency across models, hydrogen atoms and non-polypeptide entities were removed prior to analysis. All structures were spatially aligned within a common coordinate framework to enable direct qualitative comparison of global folding patterns and molecular surface architecture between study-derived and reference sequences.

Comparative assessment was conducted through visual inspection, emphasizing the conservation of overall tertiary structure and the distribution of solvent-exposed regions. This methodological framework follows the approach previously described by [40], with adaptations tailored to the specific sequences analyzed in the present study.

### Statistical analysis

Prevalence was calculated as the proportion of positive samples, and the 95% confidence interval (CI) was estimated using the Wilson score method. All statistical analyses were performed using OpenEpi [41].

## Results

### RVA detection

Between September 2020 and March 2022, a total of 240 bats were sampled at PBF, yielding 59 faecal samples, 157 rectal swabs, and 24 rectal swabs stored together with faeces in the same cryotube. All individuals appeared clinically healthy and showed no observable signs of disease. The specimens comprised 16 species distributed across 11 genera and two families. The family Phyllostomidae predominated (99.2%; 238/240), followed by Vespertilionidae (0.8%; 2/240). Most individuals were adults (93.7%; 225/240), and the sex ratio was balanced, with 120 males and 120 females (Table 1).

**Table 1:** Rotavirus A detection in Phyllostomidae bats from an urban remnant of the Atlantic Forest in Rio de Janeiro, Brazil (September 2020–March 2022), according to infection rate, age class (A: adult, S: subadult), and sex (♀: female, ♂: male)

**Subfamily**	**Specie**	Infection rate*	Age*		Sex*		**Total**
			**S**	**A**	**♀**	**♂**	
Stenodermatinae	*Artibeus lituratus*	1,9 % (2)	6	98 (2)	57 (2)	47	104
	*Artibeus obscurus*		0	6	3	3	6
	*Artibeus fimbriatus*		2	15	9	8	17
	*Platyrrhinus recifinus*		0	4	1	3	4
	*Sturnira lilium*		0	10	7	3	10
	*Sturnira tildae*		0	5	1	4	5
	*Vampyressa pusilla*		0	2	0	2	2
Carolliinae	*Carollia brevicauda*		2	4	5	1	6
	*Carollia perspicillata*	1,4 % (1)	5	64 (1)	26	43 (1)	69
Desmodontinae	*Desmodus rotundus*		0	4	3	1	4
Glossophaginae	*Glossophaga soricina*		0	3	3	0	3
Micronycterinae	*Micronycteris megalotis*		0	3	0	3	3
Phyllostominae	*Phyllostomus hastatus*		0	2	2	0	2
	*Tonatia bidens*		0	3	3	0	3
Vespertilionidae	*Myotis riparius*		0	1	0	1	1
	*Myotis nigricans*		0	1	0	1	1
TOTAL			15	225	120	120	**240**

*Positive animals are shown in parentheses

The detection rate of RVA was 1.25% (3/240; 95% exact CI: 0.42–3.6%), with three bats testing positive: two *Artibeus lituratus* (internal id numbers of V616 and V617, with Ct values of 35 and 24.5; both adult females sampled by faeces with rectal swabs), and one sample from species *Carollia perspicillata* (V968, and Ct value of 33.8, faecal sample collected from an adult male). The two RVA-positive *A. lituratus* individuals were captured concurrently at the same sampling site on the same date (February 9, 2021), whereas the *C. perspicillata* individual was captured on April 26, 2021 (Table 2).

**Table 2:**
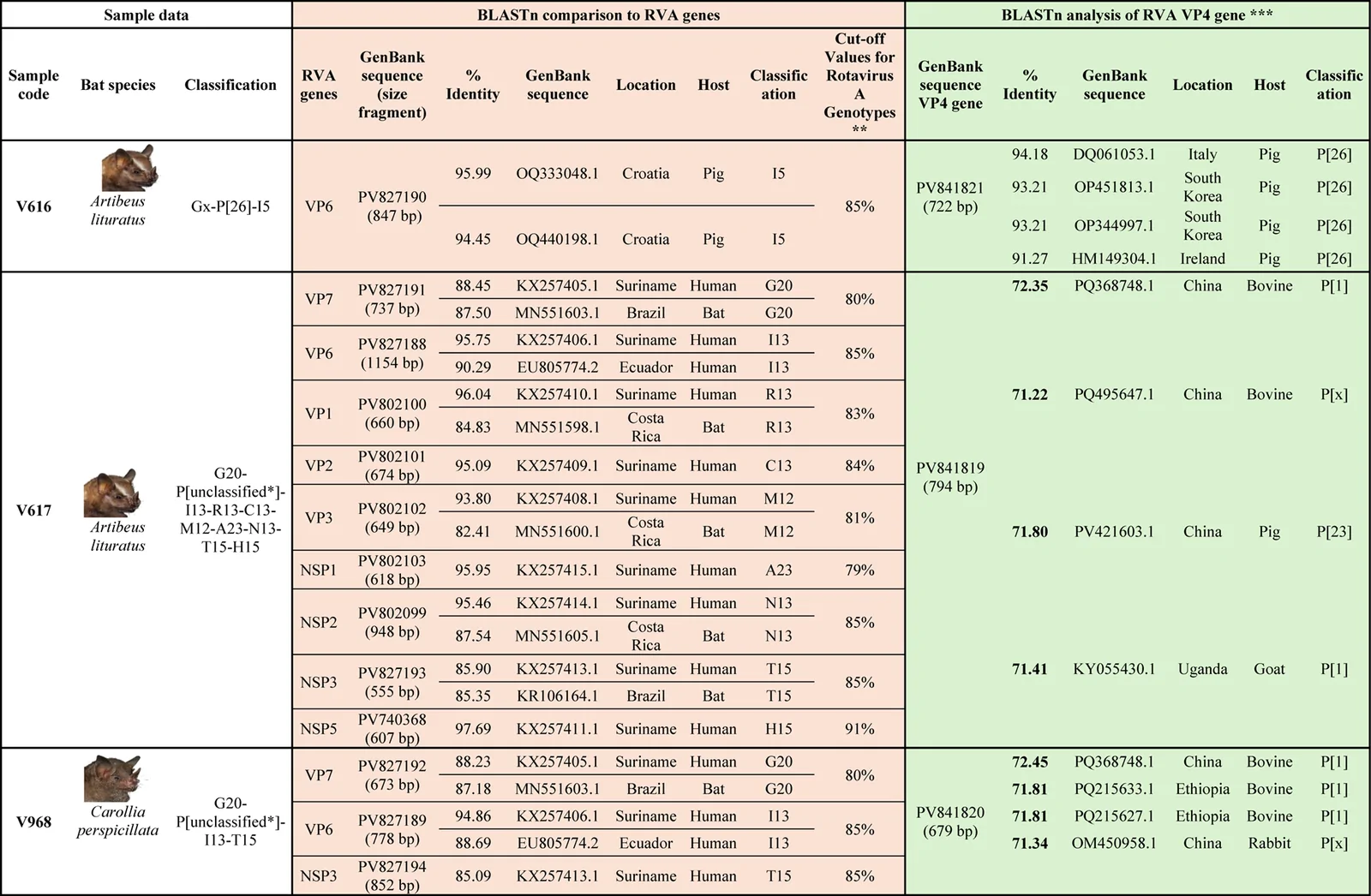
Bats positive for Rotavirus A were detected by molecular methods in an urban remnant of the Atlantic Forest in Rio de Janeiro, Brazil (September 2020–March 2022). Obtained nucleotide sequences were analyzed using BLASTn and compared with GenBank entries; VP4 gene sequences showed low similarity (<80%) to the four most closely related sequences. Photos: Roberto L. M. Novaes

* Indicate a putative novel VP4 genotype, ** according to reference [35], *** The VP4 genotype cut-off value of 80% was used based on the established criteria for Rotavirus A genotypes (reference [35]).

### RVA genotyping and phylogenetic analysis

The RVA bat constellation was investigated through whole-genome sequencing of the 11 RVA gene segments, allowing partial characterization of the three RVA-positive bat samples as follows: Gx-P[26]-I5-Rx-Cx-Mx-Ax-Nx-Tx-Ex-Hx (V616), G20-P[unclassified*]-I13-R13-C13-M12-A23-N13-T15-Ex-H15 (V617), and G20-P[unclassified*]-I13-Rx-Cx-Mx-Ax-Nx-T15-Hx (V968). Gene amplification efficiency varied among segments: VP1, VP2, VP3, NSP1, NSP2, and NSP5 where partial sequences were amplified for one sample; VP7 and NSP3 were obtained for two samples. Only the VP4 and VP6 genes were successfully identified in all three samples (Table 2), and the amplification of the NSP4 gene was unsuccessful in all samples.

For the VP7 gene segment, positive samples from *A. lituratus* (V617) and *C. perspicillata* (V968) were genotyped as G20. BLASTn showed highest (~ 88%) similarity to RVA/Human-wt/SUR/2,014,735,512/2013/G20P[28] from Suriname (GenBank accession number KX257405.1) [24] and ~ 87% similarity to a G20 strain detected from *C. perspicillata* bat species sampled in Costa Rica (GenBank accession number MN551603); (Tables 2 and 3).

**Table 3:**
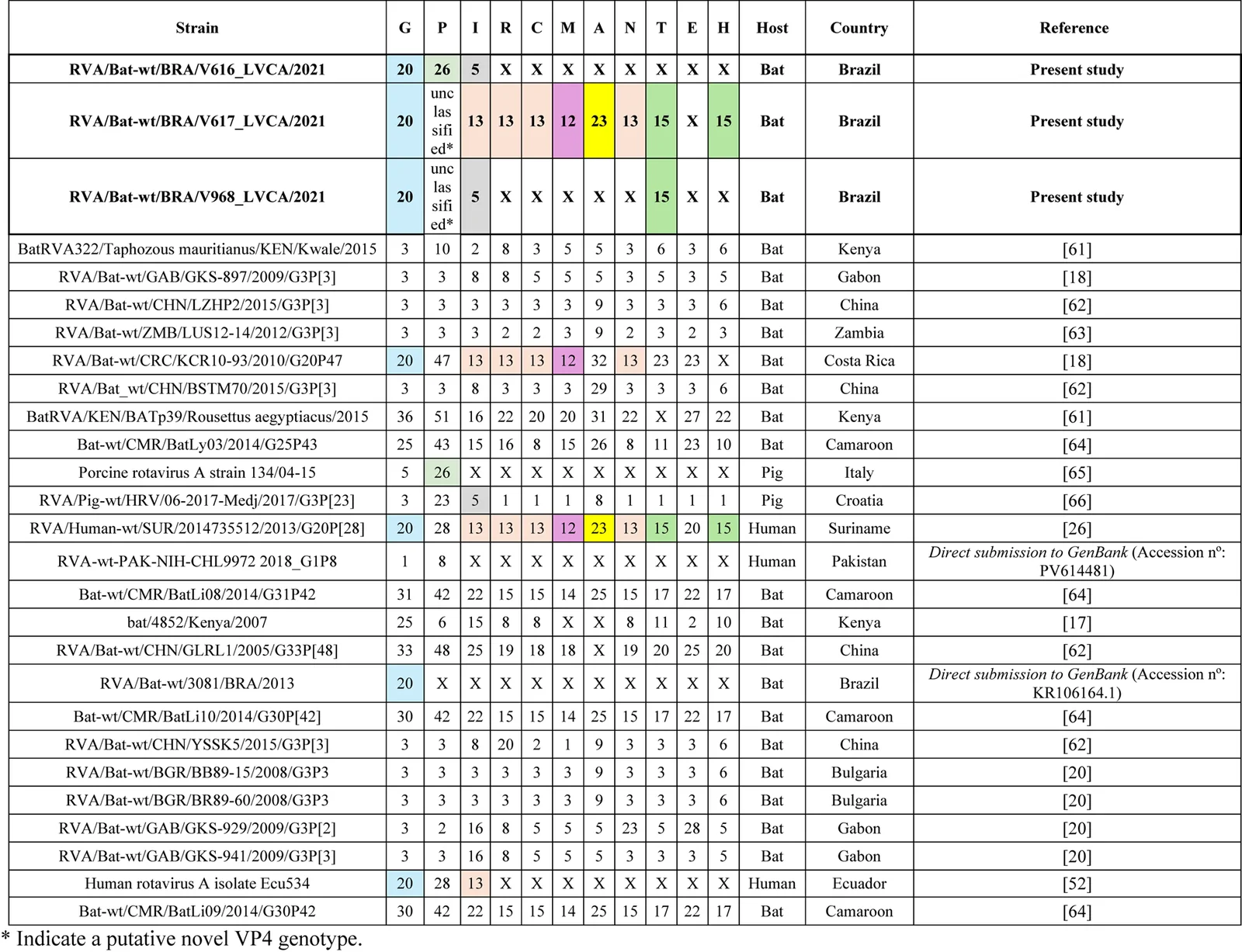
Genomic constellations of bat-associated Rotavirus A (RVA) lineages by host species. Gene segments are designated as G (VP7), P (VP4), I (VP6), R (VP1), C (VP2), M (VP3), A (NSP1), N (NSP2), T (NSP3), E (NSP4), and H (NSP5). Country of detection and NCBI accession numbers or references are indicated. Sequences from this study (bats from an urban Atlantic Forest remnant, Rio de Janeiro, Brazil, September 2020–March 2022) are shown in bold

**Fig. 2:**
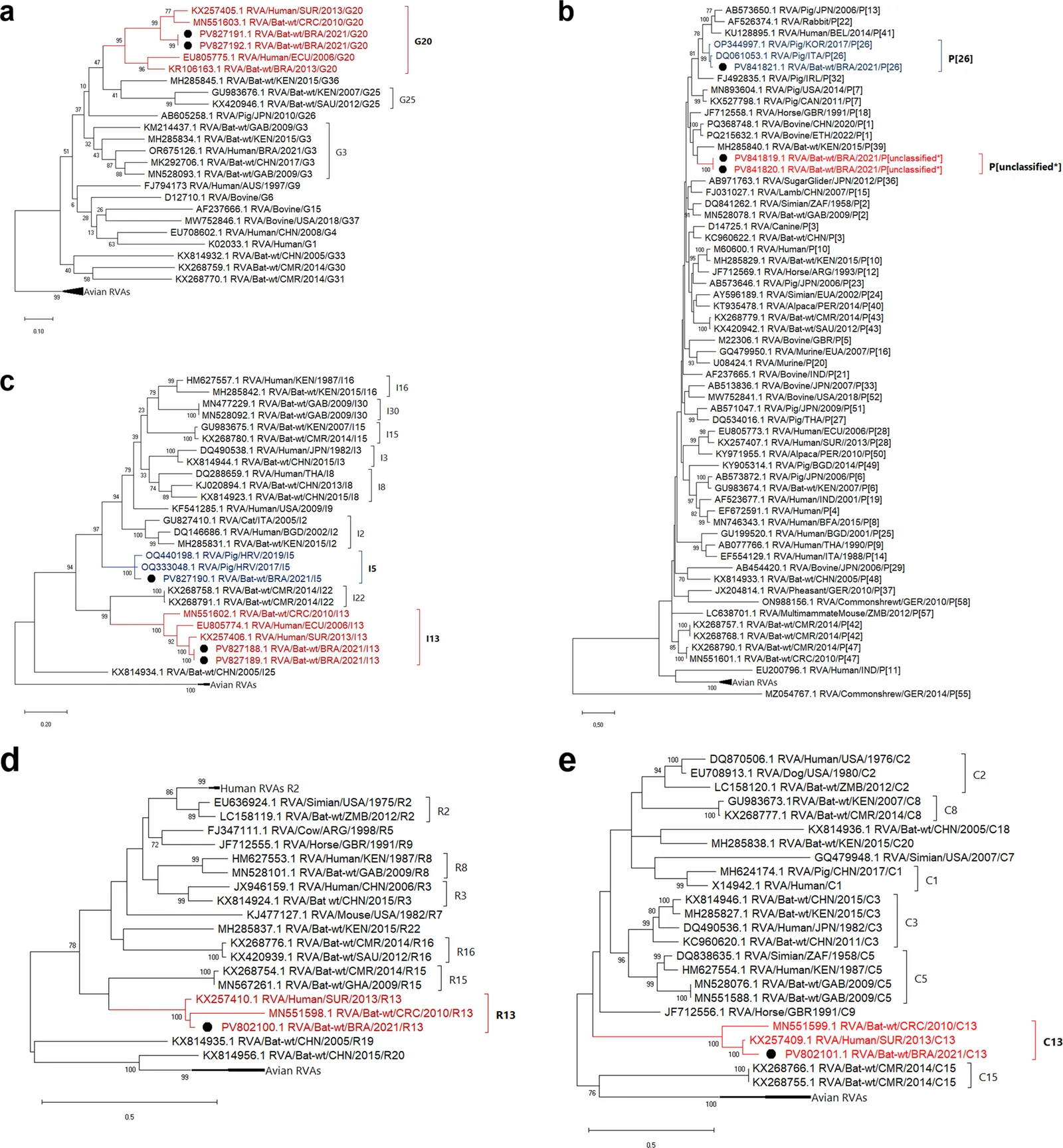

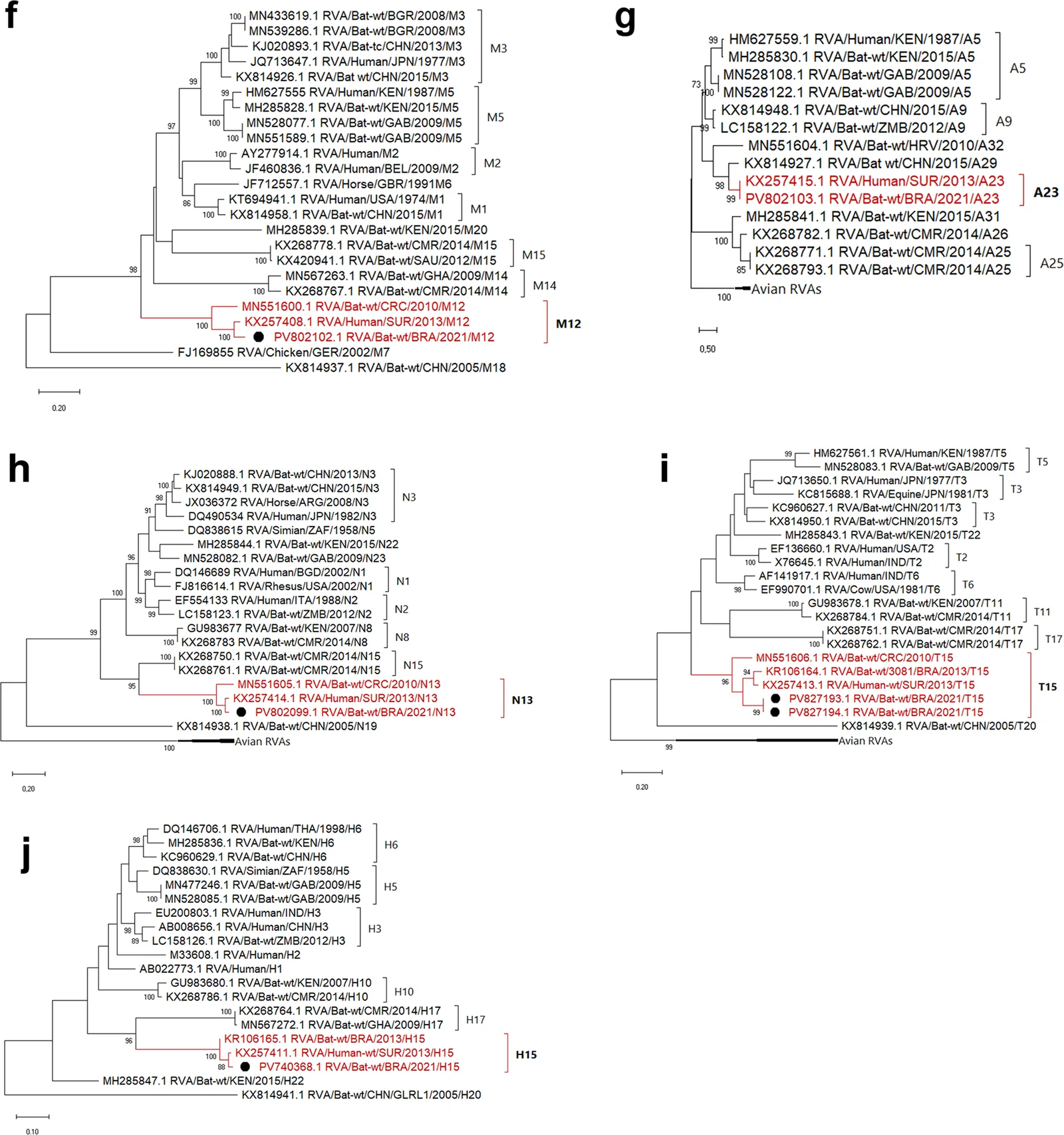
Maximum-likelihood phylogenetic trees based on nucleotide sequences of Rotavirus A genes detected in bats from an urban Atlantic Forest remnant, Rio de Janeiro, Brazil (September 2020–March 2022): VP7 (**a**), VP4 (**b**), VP6 (**c**), VP1 (**d**), VP2 (**e**), VP3 (**f**), NSP1 (**g**), NSP2 (**h**), NSP3 (**i**), and NSP5 (**j**). Trees were constructed using the General Time Reversible (GTR) substitution model for VP7, VP4, VP6, VP1, VP2, and NSP3, and the Tamura 3-parameter (T92) model for VP3, NSP1, NSP2, and NSP5. Bootstrap values above 70% are shown at the nodes. Samples obtained in this study are indicated with filled circles for clarity. Gene genotypes corresponding to the samples are highlighted in color. *Indicates a potential novel VP4 genotype

The genetic segments VP6, VP1, VP2, VP3, NSP1, NSP2, NSP3, and NSP5 of these samples showed > 80% sequence similarities to the Suriname strain RVA/Human-wt/SUR/2,014,735,512/2013/G20P[28] (Tables 2 and 3) [26]. The VP1, VP3, and NSP2 genes also shared > 80% similarity with RVA/Bat-wt/CRC/KCR10-93/2010/G20P47 from *Carollia perspicillata* sampled in Costa Rica, where as NSP3 and NSP5 genes exhibited the highest nucleotide identity (> 85%) to RVA/Bat-wt/3081/BRA/2013 from *Glossophaga soricina* in São Paulo, Brazil, which is classified as G20-P[x]-Ix-Rx-Cx-Mx-Ax-Nx-T15-Ex-H15 (Fig. 2; Tables 2 and 3) [47].

The VP6 gene was successfully genotyped in all samples. The VP6 gene from samplesV617 and V968 shared > 85% nucleotide identity with reference strains assigned to genotype I13 and clustered closely with a human RVA strain detected in a child from Suriname [26]. Sequence V616, obtained from *Artibeus lituratus*, was classified as genotype I5 and showed the highest nucleotide similarity (> 94%) to a porcine RVA strain (G3P[23]) from Croatia (Tables 2 and 3).

Furthermore, phylogenetic analyses of all sequenced genes corroborated the BLASTn results, showing that the three RVA-positive samples clustered within the same clades as the sequences with the highest nucleotide identity, thereby supporting the assigned genotypes (Fig. 2).

BLASTn analysis of the partial VP4 gene, using the 80% nucleotide identity cut-off value for VP4 genotype assignment [35] (Table 2), showed that V616 (GenBank accession number PV841821) shared high nucleotide identities, varying from 91.3 to 94.2%, with previously deposited P[26] sequences (GenBank accession numbers DQ061053.1, OP451813.1, OP344997.1 and HM149304.1). In contrast, V617 (GenBank accession number PV841819) and V968 (GenBank accession number PV841820) exhibited low identity (71.2–72.5%) with GenBank sequences. Phylogenetic analysis classified V616 within a P[26] clade containing porcine sequences identified in South Korea (GenBank accession numbers OP344997.1) and Italy (GenBank accession numbers DQ061053.1). In contrast, V968 clustered with V617 in a distinct, exclusive clade, divergent from previously reported sequences (Fig. 2).

Amino acid distance matrix analysis of the VP4 gene showed low divergence (4–7%) between the sequence obtained from the sample V616 and porcine P[26] sequences with highest BLASTn identity. In contrast, the sequences obtained from samples V617 and V968 exhibited high divergence (26–33%) relative to their closest BLASTn matches (Supplementary Table S2), corroborating the hypothesis that they may represent a candidate novel VP4 genotype, designated P[unclassified*]. Notably, the VP4 genes of the P[unclassified*] strains from V617 and V968 were highly similar to each other, with only ~ 0.9% divergence, in the nucleotide distance matrix analysis, indicating close genetic relatedness.

### In silico structural analysis of VP4 proteins

**Fig. 3:**
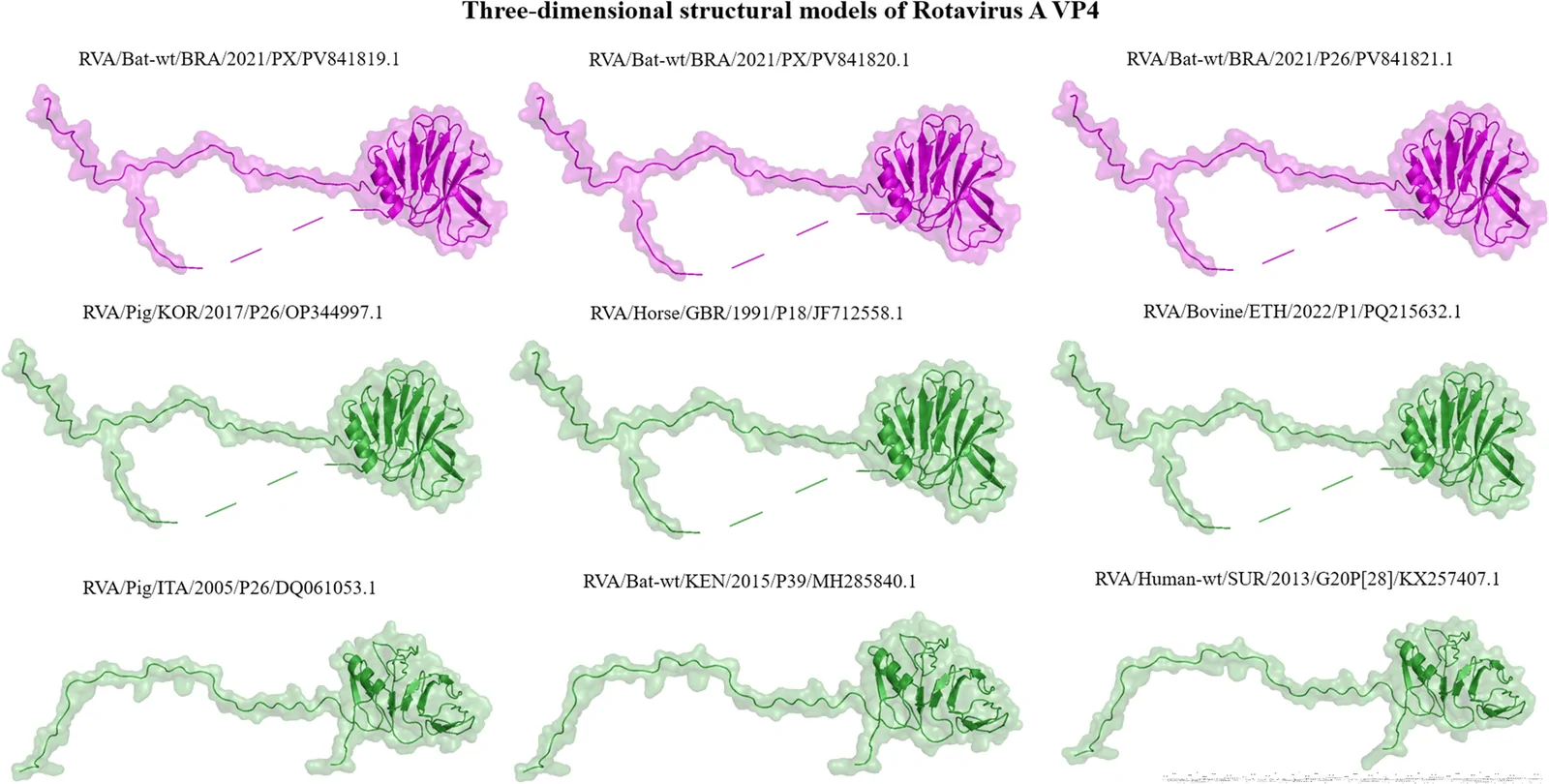
Predicted three-dimensional models of the Rotavirus A VP4 protein derived from bats from an urban Atlantic Forest remnant, Rio de Janeiro, Brazil (September 2020–March 2022), and comparison with multispecies reference sequences. Legend: The upper models (purple) correspond to the sequences analyzed in this study (PV841819.1 - P[x], PV841820.1 - P[x], and PV841821.1 - P[26]; bat origin), generated by homology modeling in Phyre2.2 using the structural template c6wxeB_ (cryo-EM reconstruction of the VP5*/VP8* assembly from rhesus rotavirus particles – upright conformation), with 100% confidence and 95% coverage. Reference models (green/forest) include DQ061053.1 (porcine - P[26]), KX257407.1 (human - P[28]) and MH285840.1 (bat - P[51]), modeled using the c8qtzA_ template (cryo-EM reconstruction of the VP5*/VP8* assembly from SA11 trypsinized triple-layered particle; 100% confidence, 94% coverage), and PQ215632.1 (bovine - P[1]), JF712558.1 (equine - P[18]), and OP344997.1 (porcine - P[26]), modeled using the c6wxeB_ template (100% confidence, 95% coverage). Prior to modeling, all protein sequences were standardized to the same length, removing non-comparable extensions in order to avoid structural artifacts resulting from absent or additional regions. Structures were visualized in PyMOL v3.1.6.1, displayed as semi-transparent surfaces with internal cartoon representation, and oriented in the same spatial reference frame to allow direct qualitative comparison of overall architecture

The structural models generated by Phyre2 showed 100% confidence and 94–95% coverage, indicating reliable predictions for most of the VP4 protein (from this study and reference). The VP4s in this study, namely: – V617 (PV841819.1); V968 (PV841820.1) and V616 (PV841821.1) – exhibited highly similar structural profiles, with nearly overlapping global folds and comparable surface topology (Fig. 3). These models also showed structural consistency with the reference sequences PQ215632.1 (P[1]; bovine), JF712558.1 (P[18] - equine) and OP344997.1 (P[26] - porcine), forming a structurally coherent group that preserved the typical VP4 architecture. On the other hand, the modeled reference sequences MH285840.1 (P[51] - bat), DQ061053.1 (P[26] - pig) and KX257407.1 (P[48] - human) shared a similar structural profile, clustering within the same conformational pattern predicted using the c8qtzA_ model. This group presented a different spatial organization of domains, although the fundamental structure of VP4 was preserved. Indeed, the analysis revealed two distinct conformational patterns (Fig. 3).

## Discussion

In this study, 240 bat individuals from 16 species were sampled, with a predominance of *Phyllostomidae*, consistent with previous surveys in the region [6, 63]. RVA was detected in three bats (1.25%) in the present study, representing a lower prevalence than that reported in bats from the Brazilian Amazon region, where RVA was detected in 18% (32/180) [29], and in bats from Bangladesh, with a prevalence of 3.8% (16/416) [43], using the same RT-qPCR target gene for detection. The prevalence observed here is also considerably lower than that reported in bats from São Paulo, Brazil (9.18%), where screening was performed using the NSP5 gene as an initial target [28]. On the other hand, the prevalence reported here is higher than that observed in a study conducted in Europe, Central America, and Africa, in which RVA was detected in 0.8% (18/2142) of bat samples using a single-round, semi-nested RT-PCR targeting the VP1 gene [18]. 

This study represents the first report of RVA genotypes G20P[unclassified*] and GxP[26] in bats from the Atlantic Forest biome and the state of Rio de Janeiro, Brazil. RVA was detected in *Artibeus lituratus* and *Carollia perspicillata*, two bat species previously identified as RVA hosts in the Brazilian Amazon [29]. These findings expand the known geographic distribution of bat-associated RVA from Atlantic Forest. Two of the three RVA-positive bats were female *A. lituratus* captured on the same day, suggesting they may belong to the same harem-based colony, a social structure characterized by one dominant male and multiple females [6, 44, 45]. Close physical contact and grooming behaviour [46], along with colony size and extensive flight ranges, likely facilitate viral transmission within and between bat populations [47–50].

To our knowledge, this is the first report of the P[26] genotype in bats, and also specifically in *A. lituratus*. The PBF region is characterized by close contact between domestic animals and wildlife, given its proximity to the Atlantic Forest and adjacent urban areas (Fig. 1). In this region, domestic animals, including subsistence pigs are maintained in households adjacent to forested areas, potentially facilitating interspecies transmission [51]. The close genetic relationship observed between the bat-derived and porcine RVA sequences suggests either a recent interspecies transmission event or a shared evolutionary origin. Reassortment events between RVA strains from bats and other hosts have been described previously; for example, some human and equine RVA strains, including the widely used reference strain SA11, may have originated from bat-associated RVA [18]. These findings highlight the potential role of bats in the ecology and dissemination of RVA genotypes among different host species [14, 18]. 

Both V617 (G20-P[unclassified*]-I13-T15) and V968 (G20-P[unclassified*]-I13-R13-C13-M12-A23-N13-T15-Ex-H15), exhibited nucleotide identities above the established cut-off values for RVA genotype classification [35] across all gene segments, except for VP4, when compared with the human strain RVA/Human-wt/SUR/2,014,735,512/2013/G20P[28] (G20-P[28]-I13-R13-C13-M13-A23-N13-T15-E20-H15), detected in a child with AGE in Suriname [26], and clustered within a well-supported phylogenetic clade that included human strains from Suriname and Ecuador and also bat strains from Costa Rica and Brazil [18, 28, 52] (Fig. 2; Tables 3 and 1).

The VP7 gene (G20) shared nucleotide similarity with a G20 strain identified in *Carollia perspicillata* from Costa Rica (G20-P[47]-I13-R13-C13-M12-A32-N13-T23-E20-Hx; GenBank MN551603). Additionally, genotype G20 has been reported in *Glossophaga soricina* and *Molossus molossus* from São Paulo State, Brazil (G20-T15-H15) [28], and in a human case of diarrhea in Ecuador (G20P[28]I13) [56] (Table 3). Esona et al. [26] proposed that the Suriname strain is likely of bat origin, and the high nucleotide identity observed with the strains detected in bats in this study is compatible with a potential scenario of interspecies transmission and possible reassortment between bat and human RVA strains. Although epidemiological links between bat and human RVA sequences have been suggested [18, 26, 28], the evolutionary and ecological dynamics underlying this relationship remain poorly understood. Collectively, these findings suggest a potential zoonotic risk associated with bat RVA strains.

In contrast, the VP4 gene sequences (GenBank accession numbers PV841819 and PV841820) displayed low nucleotide identity (< 75%) relative to all previously available RVA sequences, confirmed by high genetic distances observed in the amino acid distance matrix and below the established cut-off values for RVA genotype classification [35] (Table 2). Phylogenetic analyses demonstrated that V617 and V968 cluster within a well-supported clade (Fig. 2) and share high overall sequence similarity, with only 0.89% amino acid divergence. Taken together, these findings provide evidence supporting the proposal that the identified partial VP4 sequences may represent a novel putative VP4 genotype, designated here as P[unclassified*]. 

Structural modeling derived from in silico analyses indicated that the nucleotide divergence observed in the VP4 gene, particularly in the supposed new P[unclassified*] genotype, did not result in substantial alterations in the overall three-dimensional architecture of the analysed protein region. This finding is consistent with the hypothesis that these viruses circulate in bat populations and, therefore, must maintain structurally functional conformations compatible with viral fitness. The canonical protein structure (typically conserved three-dimensional conformation shared among homologous proteins) was maintained and highly similar to those of previously described VP4 genotypes in different host species (swine, bovine, human, equine, bat). VP4 (VP8*) is the spike protein of the RVA outer capsid and plays a central role in viral binding and entry, being proteolytically cleaved into VP8* and VP5* subunits during activation [53, 54]. It is important to emphasize that no relevant difference was observed in the VP8* domain, which mediates the initial interaction with host cell glycans [55]. Taken together, these findings suggest that the sequence mutations that define the proposed genotype mainly reflect evolutionary divergence at the sequence level, preserving the structure necessary for VP4 function, which corroborates our hypothesis that the identified bat strains may have the potential to infect other hosts, including humans.

In accordance with all the discussion above, samples V617 and V968 were classified as G20-P[unclassified*]-I13-R13-C13-M12-A23-N13-T15-H15 and G20-P[unclassified*]-I13-T15, respectively. Interestingly, although the VP4 genotype remains provisional pending successful complete sequencing, planned by our group, both strains were detected in different bat species at distinct sampling times, but originating from the same metropolitan region, suggesting local circulation and potential interspecific transmission of this new genotype. These observations highlight the importance of comprehensive analyses, including complete genome sequencing of VP4 and other viral genes, to fully characterize the evolutionary and functional properties of this genotype.

It is also worth noting that amplification of the complete genomic constellation was limited, with NSP4 and several other gene segments not recovered, likely due to low viral load, genetic divergence of bat-associated strains, and primer bias toward human RVA. The small body size of South American bats further limited available material. These constraints highlight the need for bat-specific molecular protocols and support metagenomic approaches for comprehensive genome characterization. 

Bats are widely recognized as important reservoirs of a broad diversity of viruses, including RVA, coronaviruses, lyssaviruses, adenoviruses, filoviruses, and henipaviruses, many of which are capable of infecting other animal species, including humans [6, 7, 18, 56–59]. In Brazil, in the remnants of the Atlantic Forest, bats such as *A. lituratus* and *C. perspicillata* are abundant and occupy a variety of ecotypes, including urbanized and forested environments [7, 42, 50]. They may roost in buildings and forage in areas with limited sanitation, as observed in some regions surrounding the study forest remnant, thereby facilitating contact with humans, domestic animals, and wildlife, as well as exposure to environments with poor sanitation and waste contamination. These conditions have clear implications for RVA interspecies transmission [20, 48]. The RVA-positive bats in this study appeared healthy, supporting their role as viral reservoirs. Both species form colonies, engage in mutual grooming, and travel long distances, behaviours that likely enhance viral dissemination across the region.

## Conclusions

To conclude, we identified two RVA genotypes in three free-living bats from the metropolitan of Rio de Janeiro: GxP[26], to our knowledge not detected in bats previously, and a putative novel genotype G20P[unclassified*]. The detection of a similar genotype constellation (G20-P[x]-I13-R13-C13-M12-A23-N13-T15-H15) in humans in Suriname further corroborates the zoonotic potential of bat-associated RVA strains.

Given the environmental stability of RVA and its efficient faecal–oral transmission [23, 25, 60], together with the capacity of bats such as *A. lituratus* and *C. perspicillata* to move between urban and forest environments [48, 50], enhanced RVA surveillance in wildlife is warranted, particularly in remnant forest fragments within urban landscapes. Our findings align with current knowledge on bat-associated viruses and further support the hypothesis that some RVA strains detected in humans and other animals may have originated from bats [17, 18]. The lack of continuous surveillance of RVA strains in bat populations in Brazil limits our ability to reconstruct the history of potential interspecies transmission events. These results highlight the potential role of bats in the ecology and evolution of RVA and emphasize the importance of continuous viral surveillance in both animal and human populations within a One Health framework.

## Supplementary Information

Supplementary Material 1.

## Data Availability

Partial genome sequences of bat Rotavirus alphagastroenteritidis (RVA) generated in this study have been deposited in GenBank under the following accession numbers: PV740368, PV802099–PV802103, PV827188–PV827194 and PV841819–PV841821 (Table 3). TheRVA sequences used for comparative analyses are available in GenBank under the accessions listed in Tables 2 and 3; Figs. 2 and 3 and Supplementary Table S2. All other relevant data are contained within the article and its supplementary files.
