# Supplementary material for: Identification of a putative novel rotavirus A VP4 genotype in bats from an urban Atlantic Forest, in the metropolitan region of Rio de Janeiro State, Southeastern Brazil
Source: Arch Virol. 2026 Oct 8;171(11):295. doi: 10.1007/s00705-026-06727-w (PMC13649932; doi:10.1007/s00705-026-06727-w)
Supplement: Supplementary file 1 [file 705_2026_6727_MOESM1_ESM.docx]

**Supplementary Table S1.** Primers and PCR assays used for the amplification of different genes of the rotavirus A (RVA) constellation, detected in bats from an urban Atlantic Forest remnant, Rio de Janeiro, Brazil (September 2020–March 2022). 1 F = Forward primer, R = Reverse primer; 2 = melting temperature; 3= references of the paper.

| **Gene/Name ^1^** | **Primer Nucleotide Sequence (5’-3’)** | **Type of diagnostic test** | **Tm^2^** | **PCR cycles** | **Reference ^3^** |
| --- | --- | --- | --- | --- | --- |
| **VP7** – VP7 F | ATGTATGGTATTGAATATACCAC | RT-PCR | 45^o^C | 50x | [42] |
| **VP7** – VP7 R | AACTTGCCACCATTTTTTCC |  |  |  |  |
| **VP4** – 4Con3 | TGGCTTCGCCATTTLATAGACA | One step RT-PCR | 50^o^C | 40x | [39] |
| **VP4** – 4Con2 | ATTTCGGACCATTTATAACC |  |  |  |  |
| **VP6** – GEN_VP6F | GGCTTTWAA ACGAAG TCTTC | One step RT-PCR | 50^o^C | 40x | [43] |
| **VP6** – GEN_VP6R | GGTCACATCCTCTCACT |  |  |  |  |
| **VP1** – VP1F | GGCTATTAAAGCTGTACAATGGG | One step RT-PCR | 49^o^C | 40X | [40] |
| **VP1** – VP1R | TAATCCTCATGAGAAAACACTGAC |  |  |  |  |
| **VP2** – VP2F | GGCTATTAAAGGCTCAATGGCG | One step RT-PCR | 49^o^C | 40X | [40] |
| **VP2** – VP2R | CTTCATCTTGAAATATAGCATCAC |  |  |  |  |
| **VP3** – VP3F | GGCTATTAAAGCAGTACCAGTAG | One step RT-PCR | 49^o^C | 40X | [40] |
| **VP3** – VP3 R | GTAAACATAGATTCATTACGCGGACC |  |  |  |  |
| **NSP1** – GEN-NSP1F | GGCTTTTTTTTATGAAAAGTCTTG | One step RT-PCR | 53^o^C | 40x | [43] |
| **NSP1** – GEN-NSP1R | GGTCACATTTTATGCTGCC |  |  |  |  |
| **NSP2** – GEN-NSP2F | GGCTTTTAAAGCGTCTCA G | One step RT-PCR | 50^o^C | 40x | [43] |
| **NSP2** – GEN-NSP2R | GGTCACATAAGCGCTTTC |  |  |  |  |
| **NSP3** – MAX NSP3F | GGCTTTTAATGCTTTTCAGTG | RT-PCR | 50^o^C | 40x | [43] |
| **NSP3** – MAX NSP3R | GGTCACATAACGCCCCTATAG |  |  |  |  |
| **NSP4** – Gen NSP4F | GGCTTTTAAAAGTTCTGTTCC | RT-PCR | 48^o^C | 40X | [43] |
| **NSP4** – Gen NSP4R | GGWYACRYTAAGACCRTTCC |  |  |  |  |
| **NSP4** – NSP4 sense | GGCTTTTAAAAGTTCTGTTCCGAG | RT-PCR | 48^o^C | 40X | [41] |
| **NSP4** – NSP4 antisense | GGTCACATCAAGACCATTCC |  |  |  |  |
| **NSP4** – NSP4 fw | GGAATGGCGTATTTTCC | RT-PCR | 48^o^C | 40X | [41] |
| **NSP4** – NSP4 reverse | TGTTCTTTGTAACCTGTCA |  |  |  |  |
| **NSP5** – Gen-NSP5 F | GGCTTTTAAAGCGCTACAG | RT-PCR | 48^o^C | 40X | [43] |
| **NSP5** – Gen-NSP5 R | GGTCACAAAACGGGAGT |  |  |  |  |

**Supplementary Table S2.** Genetic divergence matrix among the VP4 gene sequences of rotavirus A (RVA) obtained in bats from an urban Atlantic Forest remnant, Rio de Janeiro, Brazil (September 2020–March 2022). GenBank sequence shown in bold and the four most similar sequences retrieved from the GenBank database via BLASTn analysis. Lower divergence values (indicating higher sequence similarity) are highlighted in red. Notably, sequences PV841819.1 and PV841820.1 exhibit high divergence compared to the GenBank reference sequences, supporting the hypothesis of a putative novel VP4 genotype of RVA. Pairwise Distance Analysis in MEGA11 software.

|  | **Nucleotide** | | | | | | | | | | | | | | |
| --- | --- | --- | --- | --- | --- | --- | --- | --- | --- | --- | --- | --- | --- | --- | --- |
|  | Host | Pig/ P[26] | Pig/ P[26] | Goat/ P[1] | Rabbit/ P[x] | Pig/ P[26] | Pig/ P[26] | Bovine/ P[1] | Bovine/ P[1] | Bovine/ P[1] | Bovine/ P[x] | Pig/ P[23] | Bat/ P[unclassified*] | Bat/ P[unclassified*] | Bat/ P[26] |
|  | GenBank sequence | DQ061053 | HM149304 | KY055430 | OM450958 | OP344997 | OP451813 | PQ215627 | PQ215633 | PQ368748 | PQ495647 | PV421603 | **PV841819** | **PV841820** | **PV841821** |
| **Amino acids** | DQ061053 |  | 0,08477 | 0,52628 | 0,45858 | 0,06622 | 0,06622 | 0,49427 | 0,50759 | 0,49646 | 0,49930 | 0,47344 | 0,65386 | 0,67856 | **0,06108** |
|  | HM149304 | 0,03468 |  | 0,53237 | 0,48580 | 0,09054 | 0,09054 | 0,53959 | 0,54985 | 0,49208 | 0,49208 | 0,50604 | 0,60561 | 0,64109 | **0,09401** |
|  | KY055430 | 0,32659 | 0,32132 |  | 0,41849 | 0,50986 | 0,50986 | 0,16063 | 0,15869 | 0,17829 | 0,19504 | 0,40831 | 0,52329 | 0,54651 | 0,53078 |
|  | OM450958 | 0,29274 | 0,28264 | 0,20705 |  | 0,49267 | 0,49267 | 0,37379 | 0,38747 | 0,39066 | 0,40343 | 0,39864 | 0,56409 | 0,56125 | 0,48557 |
|  | OP344997 | 0,06656 | 0,06656 | 0,32659 | 0,30809 |  | 0 | 0,50219 | 0,50871 | 0,49299 | 0,49903 | 0,49622 | 0,63807 | 0,66167 | **0,07159** |
|  | OP451813 | 0,06656 | 0,06656 | 0,32659 | 0,30809 | 0 |  | 0,50219 | 0,50871 | 0,49299 | 0,49903 | 0,49622 | 0,63807 | 0,66167 | **0,07159** |
|  | PQ215627 | 0,33393 | 0,33393 | 0,03888 | 0,19597 | 0,34583 | 0,34583 |  | 0,01566 | 0,10499 | 0,11002 | 0,44861 | 0,49808 | 0,50687 | 0,49874 |
|  | PQ215633 | 0,34583 | 0,34583 | 0,03010 | 0,20114 | 0,35788 | 0,35788 | 0,01709 |  | 0,10007 | 0,10838 | 0,44231 | 0,49789 | 0,50666 | 0,50570 |
|  | PQ368748 | 0,32892 | 0,32367 | 0,04289 | 0,19145 | 0,32892 | 0,32892 | 0,03888 | 0,03010 |  | 0,03624 | 0,43394 | 0,49415 | 0,52084 | 0,50432 |
|  | PQ495647 | 0,33420 | 0,32892 | 0,04688 | 0,19605 | 0,33420 | 0,33420 | 0,04329 | 0,03448 | 0,00379 |  | 0,43089 | 0,51588 | 0,54353 | 0,49094 |
|  | PV421603 | 0,32892 | 0,33950 | 0,23080 | 0,22409 | 0,34484 | 0,34484 | 0,22208 | 0,21680 | 0,20067 | 0,20531 |  | 0,54318 | 0,60146 | 0,51641 |
|  | **PV841819** | 0,36266 | 0,34640 | **0,27625** | **0,30429** | 0,37365 | 0,37365 | **0,26662** | **0,27780** | **0,27884** | **0,28388** | **0,32512** |  | 0,00443 | 0,64690 |
|  | **PV841820** | 0,38566 | 0,36624 | **0,28174** | **0,31623** | 0,38566 | 0,38566 | **0,26933** | **0,28152** | **0,28030** | **0,28030** | **0,33469** | **0,00892** |  | 0,64171 |
|  | **PV841821** | **0,04255** | **0,05568** | 0,34656 | 0,31015 | **0,07346** | **0,07346** | 0,33647 | 0,34866 | 0,34484 | 0,33897 | 0,37469 | 0,38271 | 0,37914 |  |

* Indicate a putative novel VP4 genotype.
